# Identifying outcomes reported in exercise interventions in oesophagogastric cancer survivors: a systematic review

**DOI:** 10.1186/s12885-021-08290-w

**Published:** 2021-05-22

**Authors:** Louise O’Connor, Emily Smyth, Annemarie E. Bennett, Valerie Smith, Linda O’Neill, John V. Reynolds, Juliette Hussey, Emer Guinan

**Affiliations:** 1 Discipline of Physiotherapy, School of Medicine, Trinity College Dublin, Dublin, Ireland; 2 Discipline of Clinical Medicine, Trinity College Dublin, Dublin, Ireland; 3 School of Nursing & Midwifery, Trinity College Dublin, Dublin, Ireland; 4 Department of Survery St James’s Hospital and Trinity Translational Medicine Institute, Trinity College Dublin, Dublin, Ireland; 5 School of Medicine, Trinity College Dublin, Dublin, Ireland

**Keywords:** Oesophagogastric cancer, Exercise, Rehabilitation, Core outcome set

## Abstract

**Background:**

Research investigating exercise interventions in oesophagogastric cancer survivors is sparse, and the outcomes are varied. The aim of this systematic review is to identify the domains and outcomes reported in exercise interventions in oesophagogastric cancer survivors to be included in a Delphi study, with a view to informing the development of a core outcome set (COS).

**Methods:**

EMBASE, PubMed, CINHAL, Cochrane Library, SCOPUS, and PEDro were searched up to March 2020 using a predefined search strategy. The outcomes identified during data extraction were categorised using the core areas outlined in the OMERACT Filter 2.0.

**Results:**

Fourteen domains and 63 outcomes were identified. The most frequently reported outcomes were in the domains of quality of life using the EORTC-QLQ-C30 questionnaire and the relevant disease-specific modules (100%), exercise capacity/fitness/physical function (100%), anthropometrics (83.33%), physical activity (66.67%), and biomarker analysis (50%).

**Conclusion:**

This systematic review quantifies and describes the domains and outcomes examined in exercise interventions in oesophagogastric cancer survivors. Some inconsistency exists within the domains and outcomes used, and little attention was given to nutritional or economic endpoints. In order to develop a COS, a Delphi consensus process with key stakeholders is needed to identify the relevant domains and outcomes for inclusion.

**Supplementary Information:**

The online version contains supplementary material available at 10.1186/s12885-021-08290-w.

## Background

Oesophageal and gastric cancers account for nearly 9% of all cancers globally, with over 1.5 million new cases detected in 2018 [1]. Although survival rates are improving, surgical treatments for these cancers are associated with reduced physical functioning and poor health-related quality of life (QoL) [2–4]. Increasingly, exercise is being used in the management of oesophagogastric cancer, as emerging studies show that it plays a role in improving outcomes such as QoL and cardiorespiratory fitness [5, 6]. However, exercise prescription is complicated by the nutritional sequalae of the disease, including weight loss, sarcopenia, gut hormone alteration, malabsorption, and early satiety. This raises concerns that exercise training may create an excess energy deficit and contribute to further weight loss and worsening fatigue [7–12]. Therefore, interventions may need to include dietary support to ensure adequate fuel availability and replacement during and after exercise, and to manage debilitating nutritional symptoms [7, 13]. Consequently, in addition to commonly used outcomes such as QoL and exercise-related measures, it is argued that a range of other outcomes including both physical and nutritional endpoints may need to be considered in exercise interventions in oesophagogastric cancer, highlighting the value of a defined Core Outcome Set (COS) for this purpose.

A COS is defined as a standardised set of outcomes that should be measured and reported, as a minimum, in all clinical trials of a specific condition [14], thus leading to more useful trial results for data comparison and synthesis. COSs have already been developed for clinical effectiveness trials in oesophageal cancer resection surgery [15, 16], however, no COS exists for exercise interventions in oesophagogastric cancer survivors.

Little guidance exists around outcome reporting in exercise intervention trials generally, with only one relevant COS in patients with dementia under development [17]. The OMERACT Filter 2.0 [18], which was originally developed for interventions in rheumatology, provides a useful framework for identifying all key aspects, or “core areas” of a health condition, and is commonly used to aid COS development. In exercise oncology specifically, the American College of Sports Medicine Roundtable described a list of clinically relevant cancer-related health outcomes for which exercise may have therapeutic benefit, including anxiety, bone health, cardiotoxicity, chemotherapy-induced peripheral neuropathy, cognitive function, depressive symptoms, fatigue, health-related QoL, lymphoedema, pain, physical function, sleep, and treatment tolerance [7]. In addition, the 2015 Cancer and Aging Research Group NCI U13 Meeting provided recommendations for selecting outcomes for exercise intervention trials for older adults with cancer including decreased hospitalisations, reduced cancer treatment toxicity, disease prevention, disease-free survival or overall survival, and cost saving [19]. While these lists identify a considerable list of pertinent outcomes relevant to exercise interventions, there is an absence of standardisation and they lack specificity to the physical and nutritional concerns that are unique to oesophagogastric cancer survivorship.

The primary aim of this systematic review is to identify the outcomes reported in exercise interventions specifically in oesophagogastric cancer survivors to be included in a Delphi study, with a view to informing the development of a COS.

## Methods

### Search strategy

The search strategy was defined in consultation with a subject librarian. The strategy included a combination of disease terms (e.g. oesophageal cancer, oesophageal adenocarcinoma) and treatment terms (e.g. exercise therapy, physical training). Six electronic databases (EMBASE, PubMed, CINHAL, Cochrane Library, SCOPUS, and PEDro) were searched from inception to March 2020. The search strategy is available in Supporting Information 1.

### Study inclusion and exclusion criteria

Randomised and non-randomised clinical trials were included. As the purpose of this study was to identify the outcomes used in intervention trials, all study designs, including pilot and feasibility studies and published protocols were included. Studies that were not published in English and published abstracts were excluded. Studies that recruited adults (≥18 years of age) who had completed treatment with curative intent for oesophageal, oesophagogastric junction, or gastric cancer, and had been discharged from inpatient hospital care were included. Studies including aerobic and/or resistance exercise interventions to improve and/or maintain health-related physical fitness were included [20]. Studies were excluded if they used exercise modalities to improve and/or maintain skill-related physical fitness (e.g. Pilates, yoga) alone [20]. Exercise interventions could be delivered as a unimodality intervention or as part of a multimodal rehabilitation programme (e.g. exercise in conjunction with dietary counselling). Exercise interventions completed entirely during post-operative inpatient hospital stay were excluded. Studies were excluded if it was unclear as to what outcome(s) were measured.

### Study selection

Two reviewers (LOC and ES) independently screened all titles, abstracts, and full texts. Any discrepancies were resolved by discussion with a third reviewer (EG).

### Data extraction

Two researchers (LOC and ES) independently extracted relevant data using a data extraction template which was developed for the purposes of this review (Supporting Information 2). Data extracted included study characteristics, patient characteristics, intervention characteristics, and outcome characteristics. Any discrepancies were resolved through discussion.

**Fig. 1:**
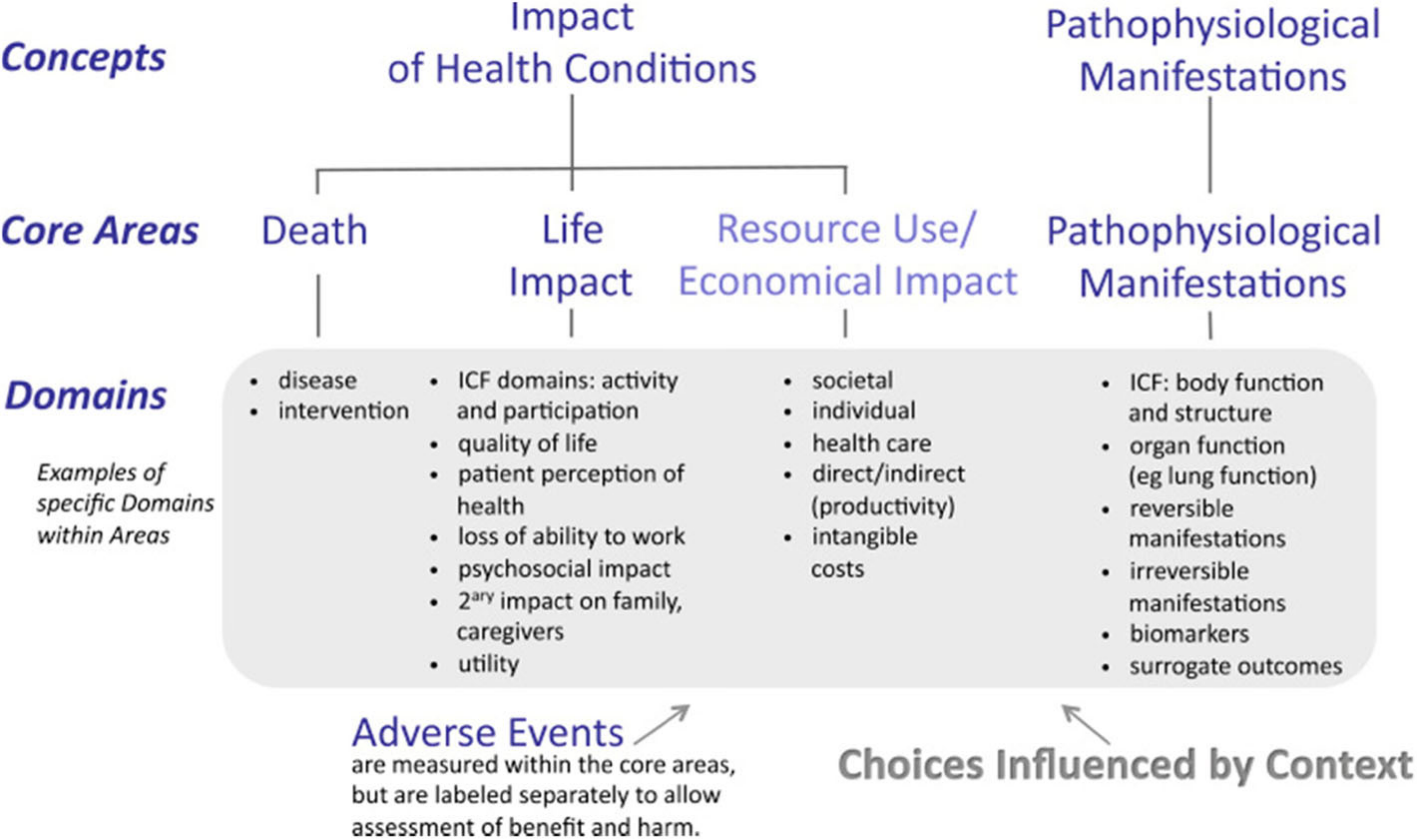
OMERACT Filter 2.0 for outcome measurement in the setting of healthcare intervention studies. Reprinted from *Journal of Clinical Epidemiology*, 67, Boers, M., Kirwan, J. R., Wells, G., Beaton, D., Gossec, L., d’Agostino, M. A., Conaghan, P. G., Bingham, C. O., 3rd, Brooks, P., Landewé, R., March, L., Simon, L. S., Singh, J. A., Strand, V., & Tugwell, P., Developing Core Outcome Measurement Sets for Clinical Trials: OMERACT Filter 2.0, Pages 745–753 [12], Copyright (2014), with permission from Elsevier

The outcomes identified during data extraction were categorised using the predetermined core areas from the OMERACT Filter 2.0 (Fig. 1) [18]. The core areas are death, life impact, and pathophysiological manifestations, and although not considered a core area, it is strongly recommended that resource use/economic impact is also included. These core areas offer useful direction, as they ensure comprehensiveness across patient-centred outcomes.

Quality assessment of outcome reporting was not examined, as the purpose of this review was purely to identify the list of reported outcomes to be included in a Delphi study, and not the quality of these outcomes, or the quality of the studies within which they were measured.

## Results

### Search results

Electronic searches identified 3564 references. After duplicate removal and title and abstract screening using Covidence systematic review management software (https://www.covidence.org/), 27 references were identified as relevant for full text review. Of these, seven studies met the inclusion criteria and were included in the systematic review. The Rehabilitation Strategies following Oesophageal Cancer (ReStOre) programme of work included one RCT [5] and one feasibility study with two papers reporting different outcomes [21, 22]. Twenty studies were excluded due to study design (*n* = 7), not being published in English (*n* = 5), study setting (*n* = 3), duplication (*n* = 2), interventions not using aerobic and/or resistance exercise modalities (*n* = 1), not oesophagogastric cancer patient populations (*n* = 1), and lack of clarity as to what outcome(s) were measured (*n* = 1). For the purpose of this review, the two papers from this one feasibility study were merged.

### Study characteristics

The study characteristics of the six included studies are outlined in Table 1. The included studies comprise three randomised control trials (RCTs) [5, 6, 24], two single arm feasibility studies [21–23], and one protocol for an RCT [25]. All included studies were published between 2017 and 2020.

**Table 1:** Study characteristics/participant characteristics

Study (author, year)	Participants (n)	Type of cancer	Cancer treatment	Mean/median time since surgery
Chang et al., 2020 [6]	88	Oesophageal	Oesophagectomy +/− neoadjuvant therapy	18 days
Cho et al., 2018 [23]	40	Gastric	Minimally invasive gastrectomy	Immediate
Fagevik Olsén et al., 2017 [24]		Oesophageal	Ivor-Lewis oesophageal resection	18.3 days (control), 19.7 days (intervention)
ReStOre Feasibility Study Guinan et al., 2017 and O’Neill et al., 2017 [21, 22]	12	Oesophageal	Oesophagectomy +/− neoadjuvant therapy	22.16 months
ReStOre RCT O’Neill et al., 2018 [5]	33	Oesophageal/oesophageal junction/gastric	Oesophagectomy or gastrectomy +/− neoadjuvant/ adjuvant/perioperative chemotherapy/chemoradiotherapy	33.68 months (control), 23.52 months (intervention)
Van Vulpen et al., 2017 [25]	150	Oesophageal	Oesophagectomy +/−neoadjuvant therapy	4 weeks to 1 year

### Participant characteristics

Four of the studies included participants who had oesophageal cancer only [6, 21, 22, 24, 25], one study included participants who had gastric cancer only [23], and one study included participants who had either oesophageal, oesophageal junction, or gastric cancers [5]. Surgery was used as a treatment technique in all studies. Mean/median time since surgery varied from immediately post-operation to 33.68 months (Table 1).

### Intervention characteristics

Exercise is defined as physical activity that is planned, structured, and repetitive, with the purpose of improving or maintaining one or more components of health-related physical fitness [26]. This review examined interventions, which prescribed aerobic and/or resistance components of exercise, and these components are described in Table 1. Study participants were supervised by physiotherapists or exercise specialists, and/or were prescribed home-based programmes following pre-intervention training by physiotherapists or exercise specialists. Three of the six studies delivered the exercise interventions as part of a multidisciplinary rehabilitation programme, which included input from other multidisciplinary team members such as nurses and dietitians [5, 6, 21, 22] (Table 2).

**Table 2:** Intervention characteristics

Study (author, year)	Study design	Country	Intervention	Comparator	Exercise type	Aerobic dose: frequency, intensity, duration	Resistance dose: frequency, intensity
Chang et al., 2020 [6]	RCT	Taiwan	Exercise, nursing education, health informatics programme	Control/usual care	Aerobic: Home-based walking programme	3–5 days/week × 12 weeks, Moderate (Borg Scale: 12–16 and 55–65% HRR), 30 mins/day or 150 mins/week	N/A
Cho et al., 2018 [23]	Single Arm, Feasibility Study	South Korea	Exercise	None	Aerobic: Walking, Resistance: Free weights, resistance bands, bodyweight exercises	Daily × 14 days, Not specified, Not specified	3 times/week × 8 weeks, Moderate (Borg Scale: 12–14)
Fagevik Olsén et al., 2017 [24]	RCT	Sweden	Exercise	Control/usual care	Resistance: Isometric, bodyweight, resistance band exercises	N/A	Daily for 3 months, 10 reps
ReStOre Feasibility Study Guinan et al., 2017 and O’Neill et al., 2017 [21, 22]	Single Arm, Feasibility Study	Ireland	Exercise, dietary counselling and education.	None	Aerobic: Treadmill-walking, stationary bike, cross trainer Resistance: Not specified	12 weeks (14 supervised sessions, 37 unsupervised sessions), Low (30–45% HRR) progressed to moderate (45–60% HRR), 20–35 min (aerobic & resistance training times combined)	12 weeks (14 supervised sessions, 37 unsupervised sessions), Light resistance work
ReStOre RCT O’Neill et al., 2018 [5]	RCT	Ireland	Exercise, dietary counselling & education.	Control/usual care	Aerobic: Treadmill-walking, stationary bike, cross trainer Resistance: Free weights, leg press, resistance bands	12 weeks (14 supervised sessions, 37 unsupervised sessions), Low (30–45% HRR) progressed to moderate (45–60% HRR), 20–35 min	12 weeks (14 supervised sessions, 37 unsupervised sessions), 2 sets of 12-RM, progressed to 6 sets of 17-RM
Van Vulpen et al., 2017 [25]	RCT Protocol	Netherlands	Exercise	Control/usual care	Aerobic: Exercise machines, Resistance: Rowing, bench press, squat, shoulder press, biceps curl, lunges, calf-raises, triceps extension, abdominal crunch	12 weeks, Weeks 1–3: 40–60% HRR, Weeks 4–8: 15–20 min at 60–70% HRR and 5–10 min at 70–89% HRR, Weeks 9–12: 10 mins at 60–75% HRR and interval training of 10 sets × 30 s maximal exercise with a 1-min active rest, 50 mins (aerobic & resistance times combined)	Twice weekly for 12 weeks, 1 set × 20–25 reps at 20-RM, progressed to 2 sets × 15–20 reps at 15-RM

*Borg Scale* Borg Scale of Relative Perceived Exertion, *HRR* Heart Rate Reserve, *mins* minutes, *N/A* Not Applicable, *Reps* Repetitions, *RM* Repetition Maximum

### Study outcomes

A total of 14 domains and 63 outcomes that aligned with the OMERACT Filter 2.0 were identified [27] (Table 3).

**Table 3:** Study outcomes

Core Area	Domain	Number of outcomes reported within domain	Number of studies reporting outcomes in domain (%)
**Mortality**	Survivorship	1	1 (16.67%)
**Life Impact**	Quality of Life	4	6 (100%)
	Physical Activity	4	4 (66.67%)
	Sleep Quality	1	1 (16.67%)
	Fatigue	1	1 (16.67%)
	Anxiety and Depression	1	1 (16.67%)
	Pain	1	1 (16.67%)
	Diet	2	1 (16.67%)
	Recurrence	1	1 (16.67%)
**Resource Use/Economic Impact**	Resource Use/Economic Impact	1	1 (16.67%)
**Pathophysiological Manifestations**	Biomarker Analysis	9	3 (50%)
	Respiratory Function	3	1 (16.67%)
	Exercise Capacity/Fitness/Physical Function	9	6 (100%)
	Anthropometrics	25	5 (83.33%)

#### Mortality

##### Survivorship

One protocol paper planned to examine this domain through the Dutch National Cancer Registry and through medical records [25].

#### Life impact

##### Quality of life

QoL was reported or planned to be reported in all six studies. All studies chose to use The European Organization for Research and Treatment of Cancer QLQ-C30 (EORTC-QLQ-C30) to measure QoL [28]. This questionnaire has a modular design with a core questionnaire and disease-specific modules. The core questionnaire contains five scales measuring function (physical, social, role, cognitive, and emotional functioning), eight symptom scales (fatigue, nausea/vomiting, pain, dyspnoea, sleep disturbances, appetite loss, constipation, and diarrhoea), financial impact, and overall QoL. In addition to the core EORTC-QLQ-C30 questionnaire [28], Chang et al. [6] and the ReStOre feasibility study [21, 22] used the oesophageal-specific module (QLQ-OES18) [29]. Cho et al. [23] used the gastric-specific cancer module (QLQ-STO22) [30] and Van Vulpen et al. [25] planned to use the oesophagogastric-specific module (QLQ-OG25) [31]. The site-specific modules take into consideration any symptoms relating specifically to upper gastrointestinal symptoms, to include xerostomia, ageusia, dysphagia, choking, indigestion, and pain on eating or drinking.

##### Physical activity

Physical activity refers to “all movement including during leisure time, for transport to get to and from places, or as part of a person’s work” and relates to physical activity levels and activity behaviour [32]. Physical activity levels were measured or planned to be measured using accelerometers in three studies [5, 21, 22, 25]. In addition to the use of accelerometers, Van Vulpen and colleagues [25] planned to further examine activity levels using exercise logbooks and the Short Questionnaire to Assess Health-enhancing physical activity (SQUASH) [33]. One study [24] used a six-level scale to establish participants’ activity levels, where low figures indicate a sedentary and a high score an active lifestyle.

##### Sleep quality

Sleep disturbance was captured in the EORTC-QLQ-C30 [28]. Van Vulpen et al. [25] also planned to capture sleep quality through the Pittsburgh Sleep Quality Index [34].

##### Fatigue

Similarly, fatigue was captured in the EORTC-QLQ-C30 [28]. Van Vulpen et al. [25] planned to use an additional tool, the Multidimensional Fatigue Inventory, to record fatigue [35].

##### Anxiety and depression

Emotional functioning was assessed in the EORTC-QLQ-C30 [28]. In addition, Van Vulpen et al. [25] planned to use the Hospital Anxiety and Depression Scale (HADS) to measure depression and anxiety [36].

##### Pain

Pain was measured using the EORTC-QLQ-C30 [28]. Fagevik Olsén et al. [24] also used a 100-mm visual analogue scale to assess pain specifically in the neck, rib cage, and shoulders.

##### Diet

Appetite loss was explored in the EORTC-QLQ-C30 [28]. Van Vulpen et al. [25] planned to explore the impact of exercise interventions on malnutrition risk using the Patient-Generated Subjective Global Assessment Short Form [37], and dietary intake using a 3-day food diary.

##### Recurrence

One study planned to measure disease recurrence using data from the Dutch Cancer Registry and through medical records [25].

#### Resource use/economic impact

##### Resource use/economic impact

Financial impact was assessed in the EORTC-QLQ-C30 [28]. For a more comprehensive insight into the economic impact of oesophagogastric cancer, Van Vulpen et al. [25] proposed to measure and value productivity losses using the iMTA Productivity Cost Questionnaire [38].

#### Pathophysiological manifestations

##### Biomarker analysis

Two studies assessed outcomes through serologic analysis [6, 21]. One study examined the effect of exercise on inflammatory status and oxidative stress [21], while another examined albumin as a measure of nutritional status [6]. One other study planned to collect blood serum, plasma and cell pellet for future analyses of biomarkers [25].

##### Respiratory function

Fagevik Olsén et al. [23] assessed respiratory function by measuring forced vital capacity, forced expiratory volume in 1 s, and peak expiratory flow, using a spirometer.

##### Exercise capacity/fitness/physical function

Exercise capacity, fitness and/or physical function refer to an individual’s ability to undertake exercise and physical tasks of everyday living [39] and were measured or planned to be measured in all studies. Five studies measured or planned to measure exercise capacity using maximal cardiopulmonary exercise tests (CPETs) [5, 6, 21–23, 25]. In addition to CPETs, Chang et al. [6] and The ReStOre feasibility study [21, 22] measured functional capacity using the distance mobilised in the Six-Minute Walk Test [40].

Grip strength is often used as an indicator of overall muscle strength and was included as an outcome to be measured using a dynamometer in three studies [23–25].

Measures of lower extremity strength and physical capacity were captured using tools such as sit-to-stand tests [23, 24]. Cho et al. [23] used the 30 Second Sit to Stand Test [41], and Fagevik Olsén et al. [24] recorded the time needed to stand 10 times from a standard chair. As well as the Sit-to-Stand Test, Fagevik Olsén et al. [24] recorded the time needed to perform 10 heel raises without support.

Flexibility was assessed by Cho et al. [23] through the back-stretch exercise and a sit-and-reach exercise, and endurance was evaluated through the wall half squat test. Range of motion was assessed by Fagevik et al. [24] using a goniometer to measure the difference in chest expansion, thoracic flexion and extension, thoracic lateral flexion, active shoulder flexion and abduction.

The Disability Rating Index was employed by Fagevik Olsén et al. [24] to assess physical disability [42]. This questionnaire comprises 12 questions, which focus on basic activities of daily life, physical activities, and work-related/more vigorous activities.

##### Anthropometrics

Five studies monitored or planned to monitor participants’ body composition throughout the exercise interventions [5, 6, 21–23, 25].

Height and weight were reported in the ReStOre feasibility study, the ReStOre RCT, and by Cho et al. [5, 21–23]. Body mass index (BMI) was recorded by Chang et al. [6] and the ReStOre feasibility study [21, 22]. Van Vulpen et al. [25] also planned to record height, weight, and BMI.

In addition, Cho et al. [23] measured the circumference of the abdomen, waist, upper arm, lower arm, hips, thigh, and calf. The ReStOre feasibility study and the ReStOre RCT [5, 21, 22] recorded waist and midarm circumference measurements, and van Vulpen et al. [25] planned to measure waist and hip circumference.

The ReStOre RCT and the ReStOre feasibility study [5, 21, 22] both examined body composition using bioimpedance analysis, which estimated fat mass, fat mass percentage, fat-free mass, fat mass index, fat-free mass index, and skeletal muscle mass. Similarly, Cho et al. [23] calculated percent body fat using the 7-site Jackson Pollock skinfold equation [43]. They also recorded subcutaneous fat thickness of chest/pectoral, midaxillary, abdominal, suprailiac, subscapular, triceps, and thigh muscles. Furthermore, they recorded values for muscle and fat volumes by obtaining muscle computed tomography (CT) cross-sectional areas of whole muscular components and fatty CT cross-sectional areas of visceral and subcutaneous fat at the levels of T11–12, L2–3, L3–4, and L4–5.

## Discussion

This review is the first stage in the development of a COS, and as such is an important information source. From this systematic review, we have identified a potential list of outcomes to be included in a Delphi study, which is the next step in developing a COS. The six studies eligible for inclusion have all been published since 2017, illustrating that this area of research is still in its infancy. This presents a timely opportunity to develop a COS.

Fourteen domains and 63 outcomes were reported in the included studies. All studies reported outcomes in the domains of QoL and exercise capacity/fitness/physical function. Less consistently reported outcomes were anthropometrics, physical activity, and blood analysis. One trial protocol planned to explore diet and nutrition as its own outcome [25]. This may be an important outcome for inclusion, as distressing bowel symptoms related to food intake, such as urgency, flatus, and diarrhoea, are commonly reported in this population [44, 45]. In a study of 100 bowel cancer survivors, 10% of participants identified physical activity as a precipitator of distressing bowel symptoms and 73% of participants restricted leisure activities in deference to the bowel symptoms they experienced [46]. Thus, it can be hypothesised that the symptoms experienced by oesophagogastric cancer survivors may have a negative impact on exercise participation, and therefore, these symptoms may require monitoring. Furthermore, reduced nutrient intake is associated with sarcopenia, which is reported in 26 to 75% of oesophageal cancer patients [47]. The European Working Group on Sarcopenia in Older People 2 (EWGSOP2) uses low muscle strength as the primary parameter of sarcopenia. A sarcopenia diagnosis is then confirmed by detection of low muscle quantity and quality using dual-energy X-ray absorptiometry (DXA), bioelectrical impedance analysis (BIA), magnetic resonance imaging (MRI), or computed tomography (CT) [48]. Only three studies in this review assessed muscle strength [23–25], and four studies measured low muscle quantity and quality using BIA or CT [5, 21–23]. These outcomes may be important measures in the identification, and consequent management, of sarcopenia, and may warrant inclusion when designing exercise interventions for oesophagogastric cancer survivors.

Research examining the cost-effectiveness of exercise interventions in some cancer populations, such as breast and colon, is emerging [49, 50], yet little is known about the economic impact of these exercise interventions in oesophagogastric cancer survivors. Although one trial protocol did plan to include the economic impact of oesophagogastric cancer on the individual [25], no study estimated the economic impact on society. This is considered important, as rising health care costs present challenges, even in wealthy societies [18, 19, 51].

In order to enhance the quality of health care to make better decisions about interventions, a COS should contain the outcomes which are most important to the stakeholders [52]. Stakeholders should comprise those who will use the COS in research, healthcare professionals with experience of patients with oesophagogastric cancer, oesophagogastric cancer survivors and/or their representatives in order to reach agreement on the outcomes to be included [53]. By including these stakeholders in the COS development process and in defining the endpoints for future studies, an interdisciplinary and collaborative approach is ensured allowing for a broad variety of relevant outcomes to be assessed. The stakeholders should also inform how and when these outcomes should be measured. The next step in this COS development process involves a Delphi consensus process with these key stakeholders [54].

## Limitations

This review has some limitations. Only studies published in English were included in this review and studies in other languages may have been inadvertently excluded. Few eligible studies were identified (*n* = 6) and one included study is a protocol, but the findings are still important in directing the next phase of this research. There was heterogeneity between included studies due to different patient characteristics and intervention characteristics. Most of the patients in this review had a diagnosis of oesophageal cancer and underwent open surgical treatment. However, some patients with gastric cancer were treated using minimally invasive surgical procedures which may result in a different rehabilitation journey. Furthermore, some participants received chemotherapy and/or radiotherapy in addition to surgery, and time since surgery differed between studies. It could be argued that these factors may have impacted the researchers’ justification of outcome choice, but the next phase of COS development will determine the salience of these outcomes. This study provides a starting point.

## Conclusion

The variation and potential gaps in outcomes reported in exercise interventions for oesophagogastric cancer survivors warrants the development of a COS, and this systematic review forms the first phase of its development. By ensuring that all interventions and reported outcomes are comprehensive in their approach, a COS would improve the quality of future research and influence oesophagogastric cancer survivorship.

## Supplementary Information

**Additional file 1: Supporting Information 1.** Search Strategy. **Supporting Information 2.** Data Extraction.

## Data Availability

All data generated and analysed during this study are included in this published article and its supplementary information files. Supplementary information files can be requested from the corresponding author (EG).

## Abbreviations

BMI: Body mass index
Borg Scale: Borg Scale of Relative Perceived Exertion
COMET: Core Outcome Measures in Effectiveness Trials
COS: Core outcome set
CPET: Cardiopulmonary exercise test
CT: Computed tomography
EORTC-QLQ-C30: The European Organization for Research and Treatment of Cancer QLQ-C30
EORTC-QLQ-OES18: The European Organization for Research and Treatment of Cancer oesophageal-specific module
EORTC-QLQ-OG25: The European Organization for Research and Treatment of Cancer oesophagogastric-specific module
EORTC-QLQ-STO22: The European Organization for Research and Treatment of Cancer gastric-specific module
EWGSOP2: The European Working Group on Sarcopenia in Older People 2
HRR: Heart rate reserve
Mins: Minutes
N/A: Not applicable
QoL: Quality of life
ReStOre: The Rehabilitation Strategies following Oesophageal Cancer
RCT: Randomised control trial
Reps: Repetitions
RM: Repetition Maximum
SQUASH: Short Questionnaire to Assess Health-enhancing physical activity
