# Supplementary material for: Identifying outcomes reported in exercise interventions in oesophagogastric cancer survivors: a systematic review
Source: BMC Cancer. 2021 May 22;21:586. doi: 10.1186/s12885-021-08290-w (PMC8141124; doi:10.1186/s12885-021-08290-w)
Supplement: Supplementary file 1 — Additional file 1: Supporting Information 1. Search Strategy. Supporting Information 2. Data Extraction. [file 12885_2021_8290_MOESM1_ESM.docx]

**Identifying outcomes reported in** **exercise interventions in oesophagogastric cancer survivors: a systematic review**

Louise O’Connor, Emily Smyth, Annemarie E. Bennett, Valerie Smith, Linda O’Neill, John V. Reynolds, Juliette Hussey, Emer Guinan

Corresponding author: Dr. Emer Guinan, School of Medicine, Trinity College Dublin, Dublin, Ireland. Email: [guinane1@tcd.ie](mailto:guinane1@tcd.ie)

Supporting Information 1 – Search Strategy:

**EMBASE**

1 'esophagus surgery'/exp

2 (esophag* NEAR/3 (surgery OR cancer OR neoplasm* OR resection* OR adenocarcinoma*)):ab,ti

3 (oesophag* NEAR/3 (surgery OR cancer OR neoplasm* OR resection* OR adenocarcinoma*)):ab,ti

4 (Gastric NEAR/3 (surgery OR cancer OR neoplasm* OR resection* OR adenocarcinoma*)):ab,ti

5 esophagectom*:ab,ti OR esophagogastroplast*:ab,ti OR esophagogastrectom*:ab,ti OR Gastrectom*:ab,ti

6 oesophagectom*:ab,ti OR oesophagogastroplast*:ab,ti OR oesophagogastrectom*:ab,ti

7 (Gastro-oesophageal NEAR/5 (tumor* OR tumour*)):ti,ab

8 (oesophagogastric NEAR/3 (surgery OR cancer OR neoplasm* OR resection* OR adenocarcinoma*)):ab,ti

9 1 OR 2 OR 3 OR 4 OR 5 OR 6 OR 7 OR 8

10 'exercise test'/exp

11 'cardiopulmonary exercise test'/exp

12 ‘experimental locomotor activity test'/exp

13 'muscle strength'/exp

14 'dynamometry'/exp

15 'isometrics'/exp

16 'ergometry'/exp

17 'physical activity, capacity and performance'/exp

18 'kinesiotherapy'/exp

19 ((exercise OR cardiopulmonary OR submaximal OR walking) NEAR/3 test):ti,ab

20 (exercise NEAR/5 (Test OR training OR program* OR intervention OR performance OR therap*)):ti,ab

21 (cpet OR cpx OR vo2 OR exercise OR strength OR treadmill OR ergometry OR bicycle):ti,ab

22 (physical NEAR/2 (activity OR performance OR capacity OR training OR fitness)):ti,ab

23 10 OR 11 OR 12 OR 13 OR 14 OR 15 OR 16 OR 17 OR 18 OR 19 OR 20 OR 21 OR 22

24 9 AND 23

**PubMED**

1 esophageal surgery[tiab] OR oesophageal surgery[tiab] OR esophageal cancer[tiab] OR oesophageal cancer[tiab] OR esophageal neoplasm*[tiab] OR oesophageal neoplasm*[tiab] OR esophageal resection[tiab] OR oesophageal resection[tiab] OR esophageal adenocarcinoma*[tiab] OR oesophageal adenocarcinoma*[tiab] OR esophagectom*[tiab] OR oesophagectom*[tiab] OR esophagogastroplast*[tiab] OR oesophagogastroplast*[tiab] OR esophagogastrectom*[tiab] OR oesophagogastrectom*[tiab]

2 "Esophageal Neoplasms"[Mesh] OR "Esophagectomy"[Mesh] OR "Esophagoplasty"[Mesh]

3 1 OR 2

4 "Exercise Test"[Mesh] OR "Muscle Strength"[Mesh] OR "Motor Activity"[Mesh] OR "Muscle Strength Dynamometer"[Mesh] OR "Exercise Therapy"[Mesh] OR "Physical Endurance"[Mesh] OR "Physical Exertion"[Mesh] OR "Oxygen Consumption"[Mesh] OR "Physical Fitness"[Mesh] OR "Ergometry"[Mesh]

5 exercise[tiab] OR cpet[tiab] OR cpex[tiab] OR cpx[tiab] OR muscle strength[tiab] OR cpet[tiab] OR cpx[tiab] OR vo2[tiab] OR walking[tiab] OR physical activit*[tiab] OR physical performance[tiab] OR fitness[tiab] OR physical endurance[tiab] OR physical training[tiab] OR resistance training[tiab] OR treadmill test[tiab] OR ergomet*[tiab] OR bicycle test[tiab]

6 4 OR 5

7 3 AND 6

**CINAHL**

1. MH "Esophageal Neoplasms/SU"
2. MH “Stomach Neoplasms/SU”
3. TI (esophag* N3 (surgery OR cancer OR neoplasm* OR resection* OR adenocarcinoma*)) OR AB (esophag* N3 (surgery OR cancer OR neoplasm* OR resection* OR adenocarcinoma*))
4. TI (oesophag* N3 (surgery OR cancer OR neoplasm* OR resection* OR adenocarcinoma*)) OR AB (oesophag* N3 (surgery OR cancer OR neoplasm* OR resection* OR adenocarcinoma*))
5. TI (esophagectom* OR esophagogastroplast* OR esophagogastrectom* OR Gastrectom*) OR AB (esophagectom* OR esophagogastroplast* OR esophagogastrectom* OR Gastrectom* )
6. TI (oesophagectom* OR oesophagogastroplast* OR oesophagogastrectom*) OR AB (oesophagectom* OR oesophagogastroplast* OR oesophagogastrectom*)
7. TI ((Gastro-oesophageal N5 (tumor* OR tumour*))) OR AB ((Gastro-oesophageal N5 (tumor* OR tumour*)))
8. TI ((oesophagogastric N3 (surgery OR cancer OR neoplasm* OR resection* OR adenocarcinoma*))) OR AB ((oesophagogastric N3 (surgery OR cancer OR neoplasm* OR resection* OR adenocarcinoma*)))
9. S1 OR S2 OR S3 OR S4 OR S5 OR S6 OR S7 OR S8
10. TI ((Exercis* N3 (test OR exam OR capacity OR cardiopulmonary OR performance OR ‘pre operative’ OR preoperative OR presurgery OR ‘pre surgery’ OR presurgical OR ‘pre surgical’) ) OR AB ( (Exercis* N3 (test OR exam OR capacity OR cardiopulmonary OR performance OR ‘pre operative’ OR preoperative OR presurgery OR ‘pre surgery’ OR presurgical OR ‘pre surgical’) )
11. TI ( 'vo2 max' OR 'aerobic capacity' OR 'anaerobic threshold' OR ‘CPET’ OR ‘CPEX’ OR ‘cpx’ OR ‘peak oxygen’ ) OR AB ( 'vo2 max' OR 'aerobic capacity' OR 'anaerobic threshold' OR ‘CPET’ OR ‘CPEX’ OR ‘cpx’ OR ‘peak oxygen’ )
12. (MH "Exercise Test") OR (MH "Exercise Test, Cardiopulmonary") OR (MH "Aerobic Capacity") OR (MH "Anaerobic Threshold") OR (MH "Therapeutic Exercise") OR (MH "Exercise")
13. S9 OR S10 OR S11
14. S9 AND S13

**Cochrane Library**

1. [mh " Esophageal Neoplasms "]
2. [mh “Stomach Neoplasms”]
3. [mh " Esophagectomy"]
4. [mh “Gastrectomy”]
5. [mh " Esophagoplasty"]
6. (esophag* N3 (surgery or cancer or neoplasm* or resection* or adenocarcinoma*)):ti,ab,kw
7. (oesophag* N3 (surgery or cancer or neoplasm* or resection* or adenocarcinoma*)):ti,ab,kw
8. (esophagectom* or esophagogastroplast* or esophagogastrectom* or Gastrectom*):ti,ab,kw
9. (oesophagectom* or oesophagogastroplast* or oesophagogastrectom*):ti,ab,kw
10. #1 OR #2 #3 OR #4 OR #5 OR #6 OR #7 OR #8 OR #9
11. [mh " Exercise Test "]
12. [mh " Muscle Strength "]
13. [mh " Motor Activity "]
14. [mh " Muscle Strength Dynamometer "]
15. [mh " Exercise Therapy "]
16. [mh " Physical Endurance "]
17. [mh " Physical Exertion "]
18. [mh " Oxygen Consumption "]
19. [mh " Physical Fitness "]
20. [mh " Ergometry "]
21. (Exercis* N3 (test or exam or capacity or cardiopulmonary or performance or pre operative or preoperative or presurgery or pre surgery or presurgical or pre surgical)):ti,ab,kw
22. ('vo2 max' or 'aerobic capacity' or 'anaerobic threshold' or ‘CPET’ or ‘CPEX’ or ‘cpx’ or ‘peak oxygen’):ti,ab,kw
23. #11 OR #12 OR #13 OR #14 OR #15 OR #16 OR #17 OR #18 OR #19 OR #20 OR #21 OR 22
24. #10 AND #23

**SCOPUS**

Esophagectomy OR Gastrectomy AND exercis*

**Pedro**

Esophageal cancer and Exercise

Gastric cancer and Exercise

Supporting Information 2 – Data Extraction

| **Study Characteristics** |  |
| --- | --- |
| Study Design |  |
| Year of Publication |  |
| Study Setting (Country) |  |
|  |  |
| **Patient Characteristics** |  |
| Number of Participants |  |
| Age |  |
| Gender |  |
| Type of Cancer |  |
| Treatment Type |  |
| Time Since Treatment |  |
|  |  |
| **Intervention Characteristics** |  |
| Intervention Type |  |
| Intervention Details |  |
| Intervention Delivery Period |  |
| Length of Intervention |  |
| Intervention Setting |  |
| Delivery Agent Type |  |
| Intervention Mode of Delivery |  |
| Comparator |  |
